# Draft Genome Sequence of *Pseudomonas* sp. Strain T2.31D-1, Isolated from a Drilling Core Sample Obtained 414 Meters below Surface in the Iberian Pyrite Belt

**DOI:** 10.1128/MRA.01165-20

**Published:** 2021-01-07

**Authors:** J. M. Martínez, C. Escudero, T. Leandro, G. Mateos, R. Amils

**Affiliations:** a Centro de Biología Molecular Severo Ochoa, Universidad Autónoma de Madrid, Madrid, Spain; b Centro de Astrobiología, Madrid, Spain; c Institute for Bioengineering and Bioscience, Instituto Técnico, Lisbon, Portugal; Loyola University Chicago

## Abstract

We report the draft genome of *Pseudomonas* sp. strain T2.31D-1, which was isolated from a drilling core sample obtained 414 m below surface in the Iberian Pyrite Belt. The genome consists of a 4.7-Mb chromosome with 4,428 coding sequences, 1 rRNA operon, 59 tRNA genes, and a 31.8-kb plasmid.

## Abstract (precis)

We report the draft genome of *Pseudomonas* sp. strain T2.31D-1, which was isolated from a drilling core sample obtained 414 m below surface in the Iberian Pyrite Belt. The genome consists of a 4.7-Mb chromosome, with 4,428 coding sequences, one rRNA operon, and 59 tRNA genes, and a 31.8-kb plasmid.

## ANNOUNCEMENT

Pseudomonas stutzeri is a Gram-negative gammaproteobacterium that has been isolated from a wide variety of environments (1). P. stutzeri is described as a facultative anaerobe that is able to denitrify (2), fix nitrogen (3), and biodegrade aromatic compounds (4). *Pseudomonas* sp. strain T2.31D-1 was isolated, using the Hungate roll tube method (5), from a strict anaerobic denitrification enrichment culture (6) of a powdered core sample (∼6 g) that was obtained 414 m below surface during the development of the Iberian Pyritic Belt Subsurface Life (IPBSL) drilling project (7) (January to March 2012) in Peña de Hierro (Iberian Pyrite Belt), Spain (37°43′45.42″N, 6°33′23.57″W).

Genomic DNA extraction from the T2.31D-1 strain was performed from a culture in Reasoner’s 2A (R2A) liquid medium using the cetyltrimethylammonium bromide (CTAB)-based method (8), and the 16S rRNA gene was amplified and sequenced as described previously (6). Quality-based editing and assembly of 16S rRNA gene reads were carried out as described previously (9). Comparison of the complete 16S rRNA gene sequence with the GenBank database (10) of the NCBI using BLAST (11) showed that the closest sequence corresponded to P. stutzeri ATCC 17588^T^ (99.93% similarity).

MicrobesNG prepared the library with the Nextera XT library preparation kit (Illumina, San Diego, CA, USA) by following the manufacturer’s protocol with the following modifications: 2 ng of DNA instead of 1 ng was used as the input, and the PCR elongation time was increased from 30 s to 1 min. The library was then sequenced using the Illumina MiSeq technology, yielding a mean coverage of 61.75× and 659,784 paired-end 2 × 250-bp reads. Trimming and quality analysis were performed using Trimmomatic v0.36 (12) and FastQC v0.11.9 (http://www.bioinformatics.babraham.ac.uk/projects/fastqc) software, respectively. *De novo* assembly was completed using SPAdes v3.14.1 (13). Extrachromosomal genetic elements were assembled using Recycler (14). Mauve Aligner v2.4.0 (15) was used to align plasmid contigs against the chromosomal assembly. Contigs were ordered in scaffolds using SSPACE software (16). Unless otherwise specified, default parameters were used for all software. A 4,762,555-bp chromosome was obtained in 47 scaffolds with an *N*_50_ value of 282.20 kb and a GC content of 63.90%, similar in size and GC content to other sequenced P. stutzeri strains (1, 4, 17–19). In addition, this strain harbored one plasmid of 31,837 bp assembled in two scaffolds, similar to other strains of this species (20).

Prokka v1.14.5 software (21) and the RAST platform (22) were used for gene prediction and annotation, using P. stutzeri ATCC 17588 as a genome reference. A total of 4,428 coding DNA sequences, 1 rRNA operon, a second copy of a 5S rRNA gene, 59 tRNAs, and 1 transfer-messenger RNA were identified. Genes involved in denitrification, thiosulfate oxidation, phosphite oxidation, catabolism of aromatic compounds, fermentation, and heavy metal (Cd, Co, Cr, Cu, and Zn) resistance were detected in the chromosome.

### Data availability.

Reads were deposited in DDBJ/ENA/GenBank under the accession number ERR3773731 and the complete genome sequences and annotations under the accession number CAJFAG010000000 for the chromosome (annotation under the accession number GCA_903995555) and the accession number CAJFAF010000000 for the plasmid (annotation under the accession number GCA_903995525). All of the reads and sequences are included under the study number PRJEB35933.
